# Bilateral Hypoglossal Nerve Palsy After Occipitocervical Fusion

**DOI:** 10.5435/JAAOSGlobal-D-19-00127

**Published:** 2020-05-18

**Authors:** Emmett J. Gannon, Chris A. Cornett

**Affiliations:** From the Department of Orthopaedic Surgery and Rehabilitation, University of Nebraska Medical Center, Omaha, Nebraska.

## Abstract

A 63-year-old man sustained a Jefferson fracture and was treated nonoperatively by a separate treating surgeon. Because of the symptomatic malalignment and nonunion after 6 months of nonsurgical management, the patient was seen for a second opinion. Occiput to C3 arthrodesis was performed. Postoperatively, the patient was diagnosed with a bilateral hypoglossal nerve palsy. Hypoglossal nerve injuries after cervical spine fractures and posterior cervical procedures are a very rare occurrence. This is the first case report of a bilateral hypoglossal nerve palsy following occipitocervical arthrodesis.

A deficit of the hypoglossal nerve is a very rare postoperative complication. More commonly, it presents as a sign of an underlying pathologic condition, such as a tumor or stroke.^1^ Hypoglossal nerve palsies present as dysphagia, dysarthria, and ipsilateral tongue deviation in unilateral cases. There have been limited reports of hypoglossal nerve palsies after anterior or posterior cervical spine surgery, all being unilateral deficits.^2,3,4,5,6,7^ Ames et al performed a multi-institutional study with a goal of identifying the incidence of hypoglossal nerve palsies after cervical spine surgery. This study identified one palsy which resulted in an overall incidence of 0.01%.^8^ We present a bilateral hypoglossal nerve palsy after occipitocervical fusion.

## Case Report

**Figure 1:**
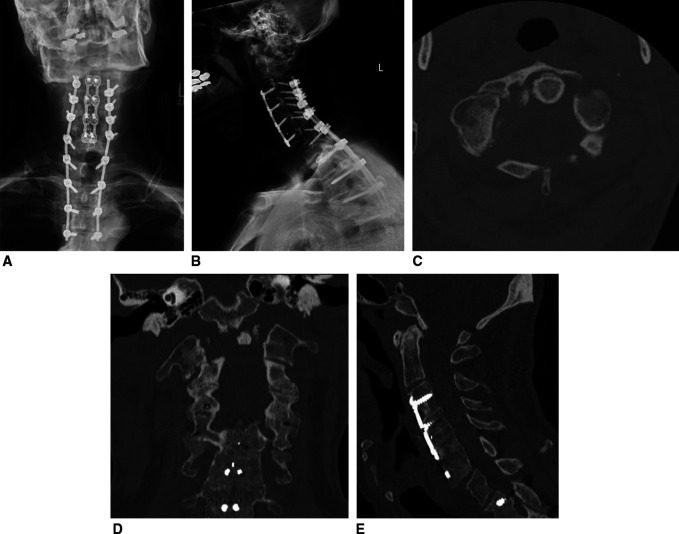
Radiograph demonstrating **A**, AP and (**B**) Lateral x-rays of the patient's cervical spine demonstrating a displaced and comminuted C1 burst fracture with nonunion. Previous C3 to C6 ACDF done a few years ago and C3-T4 posterior cervical fusion done 6 months earlier for a C6 facet fracture also shown. **C**, Axial (**D**) coronal, and (**E**) sagittal CT images of the patient's fracture, demonstrating right-sided displacement of C1 with cranial settling and posterior displacement of the occiput with fracture nonunion of C1. ACDF = anterior cervical discectomy and fusion

**Figure 2:**
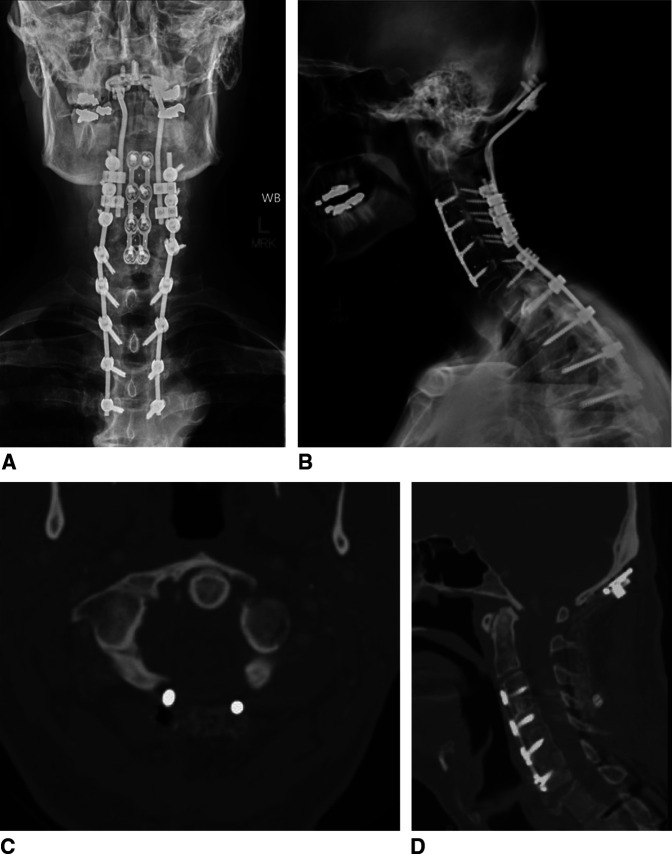
Radiograph demonstrating **A**, AP and (**B**) Lateral upright postoperative cervical x-rays after occipitocervical fusion with extension to previous instrumentation and fusion with improved alignment. **C**, Axial and (**D**) Sagittal images obtained from the patient's postoperative CT angiography demonstrates the known C1 fracture nonunion with no significant change in the cranial settling or posterior displacement of the occiput.

A 63-year-old man presented for a second opinion on the management of a comminuted C1 burst fracture he had sustained after a fall down a flight of stairs before 6 months. Of note, the patient had a history of an uncomplicated C3 to C6 anterior cervical disectomy and fusion for radiculopathy done by an outside surgeon a few years before sustaining this injury. In addition to the Jefferson fracture, the patient also sustained an unstable C6 facet fracture. This was treated with a C3-T4 posterior decompression and instrumented fusion two days after his initial injury by a separate surgeon. At that time, the Jefferson fracture was treated nonsurgically in a cervical collar. The decision to treat only the C6 fracture was because of the patient's request despite the recommendation of surgical intervention by the initial treating surgeon. Over the next 6 months, follow-up imaging noted continued nonunion and increased right-sided displacement (Figure 1). The patient was seen for a second opinion because of his continued pain and torticollis and was subsequently offered surgical intervention. The patient was admitted the day before surgery and placed in 15 pounds of cervical traction with the goal of attaining improved alignment before surgery. On the day of surgery, anesthesia was induced and video-assisted laryngoscopy was used to attain endotracheal intubation. No difficulties were noted during the intubation. The patient was then transferred to the operating table and placed in the prone position. Using a posterior approach, occiput to C3 posterior spinal fusion was done (Figure 2). Connectors were used at C3, C4, and C5 to attach rods from the occipital plate to the previously placed construct. Owing to the chronicity of the patient's malalignment, the cervical traction was the sole method of reduction. The Gardner-Wells tongs were then removed, and the patient was extubated and transferred back to the hospital bed. The patient was noted to have been intubated for a total of 183 minutes. No complications were encountered intraoperatively, and the patient remained hemodynamically stable throughout the perioperative period. Approximately 2 to 3 hours postoperatively, the patient was observed to have developed significant dysarthria and difficulty swallowing. On further evaluation, the patient was also noted to be unable to protrude his tongue. A neurologist was consulted, and the patient was diagnosed with an isolated bilateral hypoglossal nerve palsy. Perioperative MRI imaging of the patient's brain and neck along with both a head and neck computed tomography angiography (CTA) were reviewed and were negative for any identifiable cause including vascular abnormalities, compressive pathology, or entrapment of the nerve in the fracture.

Owing to the patient's dysphagia, speech therapy and a nutritionist were consulted to provide and assess the safest method for the patient to attain adequate nutrition. After a thorough evaluation, the patient was deemed able to attain sufficient nutrition with nectar consistent foods administered with a syringe.

Although discussing the patient's diagnosis, the patient recalled he had sustained a similar injury after his C3-T4 posterior spinal fusion that had lasted only a few days. Per the patient, he developed mild dysarthria and difficulty swallowing, which he thought only affected the right side of his tongue. These symptoms completely resolved over the next few days after the initial onset. In retrospect, this may have been an unilateral palsy secondary to airway management or because of continued fracture displacement.

The patient was discharged on postoperative day 5. He made slow improvements over the next several weeks. At 6 months postoperatively, the patient's speech had significantly improved; however, he still had some residual difficulty with swallowing and required continued speech therapy on a weekly basis.

## Discussion

A hypoglossal nerve palsy is a rare postoperative complication. There have been reported cases of unilateral deficits after both anterior and posterior cervical spine surgery, although no bilateral deficits after cervical or occipitocervical surgery have been reported.^3,4,5,6,7^ Only four cases of postoperative bilateral hypoglossal nerve palsies have been previously reported, all being thought to have been a complication from perioperative airway management.^9,10,11,12^ Mechanisms related to intubation include compression from the endotracheal tube during the procedure, pressure to the tongue's lateral roots from the laryngoscope during intubation, or cricoid pressure done to assist with intubation.^9,13,14^ It is also believed to be increased in posterior cervical cases because of the flexion of the neck necessary for exposure. This would then result in an endotracheal tube that may be at higher risk of causing compression of the hypoglossal nerve because it is in closeness, proximity to the larynx. Hyperextension is also thought to be a potential risk factor because it may result in stretching of the nerve as it courses along the lateral aspect of the transverse process of the atlas.^15^ This hyperextension may occur because of the positioning or during intubation. Because the duration of surgery has a direct relationship with the length of compression, this is thought to contribute to worsening injury.^16,17^

Although there have been multiple reports of hypoglossal nerve palsies thought to have resulted from airway management, there have been very limited reports of patient's sustaining cranial nerve injuries as a result of Jefferson fractures. Connolly et al^18^ reported a case in which a patient sustained a Jefferson fracture that resulted in lesions of the hypoglossal nerve, along with cranial nerves IX, X, and XI. This unilateral lesion of the cranial nerves IX, X, XI, and XII is commonly referred to as Collet-Sicard Syndrome. Similarly, Zielinski et al^19^ reported a C1 burst fracture that resulted in palsies of cranial nerves IX, X, and XII. Zielinski et al hypothesized that the Jefferson fracture could cause the nerve injuries because of the limited space between the transverse process of C1 and the styloid process, in which the cranial nerves IX, X, XI, and XII travel through. An anatomic study of cadavers by Ebraheim et al revealed that the hypoglossal nerve exits the skull through the hypoglossal foramen and after coursing caudally it travels ventral to the C1 lateral mass and the facet joint of C1 to C2.^20^ The nerve lies approximately 2 to 3 mm lateral to the midanterior aspect of the lateral mass of C1. Lateral displacement of the atlas combined with cranial settling can result in compression of the nerves between the transverse process of C1 and the styloid process or the stylohyoid ligament. Regarding our case, this relationship may have resulted in right-sided stretch and contralateral compression during the correction perioperatively. In addition, there was considerable cranial settling and posterior displacement of the occiput noted both in the preoperative and postoperative imaging (Figures 1, E and 2, D). This displacement likely led to compression and stretching of the nerve, which would have placed the nerve at a significantly higher risk of injury. The original injury and subsequent displacement could also be an explanation for the temporary palsy the patient had experienced postoperatively after his C3-T4 fusion.

In the case of a bilateral nerve palsy, it is important to consider the affect the palsy may have on attaining adequate nutrition. In some instances, it may be necessary to attain an alternate route of nutrition. Overall, the incidence of hypoglossal nerve palsy is a very rare occurrence in cervical spine surgery. The cause of injury in our case was likely secondary to the patient's Jefferson fracture with chronic worsening malalignment and subsequent correction with perioperative airway management also being a possible cause. This case highlights the importance of identifying postoperative cranial nerve deficits because this patient's history of a previous palsy likely placed him at a higher risk of developing a subsequent palsy. One consideration would be the utilization of intraoperative neuromonitoring for patient's considered at higher risk. There have been reports of intraoperative hypoglossal nerve monitoring being beneficial during brain and head and neck surgery.^21,22^ Intraoperative monitoring may help identify an early insult and potentially prevent or decrease the severity of injury. However, with this complication being such a very rare occurrence, the routine utilization of intraoperative monitoring would likely not be cost-effective.
